# LOX-mediated adipose tissue stiffening promotes weight rebound

**DOI:** 10.1016/j.jlr.2026.101121

**Published:** 2026-08-11

**Authors:** Shanshan Zhu, Shuo Yang, Yuxin Jin, Xinwen Yu, Xin Wang, Aili Yang, Duolin Niu, Guohong Zhao, Bin Gao

**Affiliations:** 1 Department of Endocrinology, Tangdu Hospital, Fourth Military Medical University, Xi’an, P.R. China; 2 The Key Laboratory of Biomedical Information Engineering of Ministry of Education, School of Life Science and Technology, Xi’an Jiaotong University, Xi’an, P.R. China

**Keywords:** weight rebound, biomechanics, adipose tissue stiffness, lysyl oxidase, lipid accumulation

## Abstract

Weight rebound remains the core challenge in the long-term management of obesity. Although current mechanistic understanding mainly focuses on physiological adaptations, the role of biomechanical factors remains largely unexplored. This study aims to investigate the contribution of lysyl oxidase (LOX)-mediated adipose tissue (AT) stiffening to the process of weight rebound. Our clinical analysis first established a correlation among obesity, elevated AT stiffness, and higher LOX levels. We then demonstrated that the weight rebound model mice displayed accelerated lipid accumulation and worsened metabolic functions. Importantly, AT stiffness dynamically increased during obesity, failed to normalize after weight loss due to persistent fibrosis, and reached its highest level during the regain stage. LOX expression followed an identical temporal pattern, showing a strong positive correlation with tissue stiffness. Mechanistically, mimicking a high-stiffness microenvironment in vitro promoted adipocyte differentiation and lipid accumulation in a LOX-dependent manner. Crucially, pharmacological inhibition of LOX in vivo significantly attenuated the rate of weight rebound. Our findings provide supportive evidence that LOX-mediated AT stiffening creates a pro-adipogenic mechanical microenvironment that contributes to accelerated weight rebound and metabolic deterioration, highlighting the LOX-stiffness axis as a potential therapeutic target for preventing weight rebound.

Obesity is a chronic disease that endangers health. Its incidence rate is increasing year by year worldwide and has become the sixth major risk factor for death and disability in China (1). In recent years, guidelines, consensus, and authoritative journals both domestically and internationally have emphasized the importance of long-term weight management for obese patients (2). Although short-term weight loss can be achieved through lifestyle interventions, drug treatment, and weight loss surgery (3, 4), but over time, weight can easily increase again, leading to weight rebound. Clinical studies have revealed a frustrating reality: the vast majority of obese patients regained more than 80% of their weight loss within 5 years after weight loss (5). Another study has shown that about 60% of obese patients experience repeated weight loss, but it is difficult to regain normal weight (6). This “yo-yo effect” not only offsets the metabolic benefits of weight loss, but also exacerbates AT dysfunction (7), glucose intolerance (8), insulin resistance (9), and the risk of cardiovascular disease (10), when compared with obesity. Therefore, clarifying the driving mechanism of this rapid rebound is the core of achieving sustainable weight management.

Currently, the understanding of weight rebound mainly focuses on physiological adaptations, such as reduced energy expenditure, reduced oxidative decomposition and dysregulation of appetite hormones (*e.g.*, ghrelin and leptin) (11, 12). Although fat inflammation and macrophage infiltration are believed to generate immunological “obesity memories” (13, 14), these soluble factors are relatively short-lived and volatile. A key question still remains: Is there a more persistent mechanical change within AT that locks the tissue in a state of easy rebound? Although AT is recognized as a dynamic endocrine organ, the role of its structural scaffold-extracellular matrix (ECM) in carrying this “memory” has not been explored.

In recent years, with the continuous development of biomechanics, more and more research has begun to focus on the regulation of cell fate and function by the cellular mechanical microenvironment (15, 16). AT is not merely a passive energy storage depot, but a dynamic and complex endocrine organ. In healthy AT, the loose ECM grid composed of collagen and fibronectin provides structural support for adipocytes and regulates the activity of growth factors and signaling molecules (17). During the process of obesity, AT dynamically expands through adipocyte hypertrophy or proliferation, requiring continuous remodeling of the ECM to adapt to this expansion. However, when the threshold exceeds the buffering capacity of adipocytes, excessive ECM components deposit, leading to fibrosis of AT (18). In addition, hypoxia and inflammation caused by obesity can further upregulate the expression of ECM related genes, leading to increased fibrosis of AT (19, 20). The increased fibrosis of AT during obesity is not merely a bystander effect, but an active biomechanical signal that can profoundly influence cell behavior, a process known as mechanotransduction. The process can affect the proliferation, differentiation, and lipid uptake function of adipocytes, or cause AT dysfunction (21, 22). However, there is still a lack of characterization of the biomechanical properties of AT and its functional contribution to weight rebound.

Lysyl oxidase (LOX) is an ECM remodeling enzyme that catalyzes covalent cross-linking of collagen and elastin, regulating the stiffness of the ECM and altering the mechanical microenvironment of cells (23, 24). LOX expression is upregulated in various fibrotic diseases and has been found to be closely related to metabolic dysfunction caused by obesity (25, 26). We hypothesize that LOX plays a pivotal role in weight rebound by creating a persistent, pro-adipogenic mechanical microenvironment. Even after weight loss, LOX-mediated, irreversible ECM cross-linking may prevent AT stiffness from normalizing. And the residual stiffness creates a persistent microenvironment that promotes lipid accumulation, thereby accelerating weight rebound upon re-exposure to caloric surplus. However, there is a critical gap in our understanding of how the mechanical properties of AT act in weight rebound.

Herein, we attempted to investigate how LOX-mediated AT stiffening creates a persistent pro adipogenic mechanical microenvironment that drives accelerated weight rebound and metabolic deterioration. Specifically, we firstly established the relationship between BMI, AT stiffness, and LOX expression levels. We then characterized the dynamic changes in AT stiffness and LOX expression in different stages of weight rebound in mouse model. Furthermore, we investigated the effects of LOX on adipocyte differentiation ability and lipid accumulation levels in vitro. Finally, we verified the therapeutic potential of LOX inhibition in attenuating weight rebound in vivo.

## Materials and methods

### Cell culture

3T3-L1 cells were cultured in high glucose DMEM at 37°C under 5% CO_2_ atmosphere. The medium was supplemented with L-glutamine (2 mM), sodium pyruvate, penicillin (100 units mL^−1^), streptomycin (100 μg mL^−1^), and 10% FBS.

### Hydrogel preparation

Commercialized methacryloyl gelatin (GelMA, EFL-GM-30, and EFL-GM-60) was purchased from EngineeringForLife. Firstly, 20 ml PBS was added to the brown glass bottle containing initiator LAP and dissolve at room temperature for 30 min. Then, 50 mg EFL-GM-30 and 75 mg EFL-GM-60 were added into 2 ml LAP solution, and dissolved at 37°C in water bath, obtain 5% GelMA-30% and 7.5% GelMA-60 solution, respectively. Later, The solution was filtered by 0.22 μm filter, and was added into 48 well plate with 200 uL per well. Finally, the sample wells were irradiated with 405 nm light source for 60 s to prepare hydrogels with different stiffness.

### Mechanical characterization of hydrogel

To characterize the mechanical properties, hydrogels with 2 mm thickness and 8 mm diameter were prepared and soaked in DMEM for at least 24 h to allow swelling. Rheology measurements were then taken with a rotational rheometer (Anton Paar MCR 302, Austria) using a 8 mm stainless steel parallel plate on a temperature-controlled Peltier system at 37°C. At the beginning of the test, the plate slowly descended until a normal force of ∼20 mN was reached. The storage modulus was measured by a frequency sweep test performed at a strain of 0.1% and frequency from 0.1 rad s^−1^ to 10 rad s^−1^.

### Cell viability

To evaluate the viability of 3T3-L1 after culturing on hydrogels, Calcein/PI Cell Viability Assay Kit (Beyotime, C2015M, China) was used. Samples were incubated with 1 ml testing buffer containing 1 μl Calcein and 1 μl PI for 30 min at 37°C under 5% CO_2_ atmosphere, followed by three washes with PBS. Imaging was performed with laser scanning confocal microscope (LSCM, OLYMPUS IX81). Live and dead cells were counted by using a cell counter plug-in for ImageJ.

### Methyl thiazolyl tetrazolium assay

5,000 3T3-L1 cells were seeded in each well of 96-well plates and the BAPN with various concentrations were added and cultured for 48 h. The final absorbance was detected to assess cell viability using a Bio-Rad 680 microplate reader.

### Differentiation of 3T3-L1

Firstly, 2 × 10^4^ 3T3-L1 cells were seeded in each well of 24-well plates and cultured until the cells reached 100% confluence. Secondly, the cells were incubated as a confluent culture with the Pre-adipocyte Expansion Medium (90% DMEM and 10% Bovine Calf Serum) for another 48 h. Thirdly, the growth medium was replaced with an identical volume of Differentiation Medium (90% DMEM, 10% FBS, 1.0 μM dexamethasone, 0.5 mM IBMX, and 1.0 μg/ml insulin) and incubated for 48 h. Fourthly, the Differentiation Medium was replaced with Adipocyte Maintenance Medium (90% DMEM, 10% FBS, and 1.0 μg/ml insulin). Finally, the Adipocyte Maintenance Medium was replaced every 48–72 h, and the cells should be fully differentiated between 7 to 15 days after induction, as evidenced by observation of lipid droplet formation.

#### Hydrogel culture

3T3-L1 cells were seeded onto hydrogels at the pre-adipocyte stage and maintained on hydrogels throughout differentiation. The later differentiation steps were the same as previous.

#### BAPN treatment

BAPN (1 mM) was added to 3T3-L1 cells simultaneously with the corresponding medium, and refreshed every 48–72 h along with the medium change and maintained throughout the entire differentiation period (including pre-differentiation stage, differentiation stage and maintenance stage). This timeline ensures the presence of LOX inhibition during the active stages of lipid accumulation and ECM deposition.

#### siRNA knockdown

3T3-L1 pre-adipocytes were transfected with siRNA of LOX two days before differentiation induction, and the knockdown efficiency was confirmed by qRT-PCR and Western blot before differentiation analysis.

### BODIPY staining for lipid accumulation quantification in 3T3-L1

To quantify intracellular lipid accumulation after culture on hydrogels with different stiffness, BODIPY 493/503 (TargetMol, 121207-31-6, USA) was used to label lipids. Firstly, the hydrogels were first fixed with 4% paraformaldehyde for 10 min, and the samples were washed with PBS three times time for 5 min each. Then, samples were permeabilized with 0.2% Triton X-100 for 15 min at room temperature, and washed three times with PBS for 5 min each. Later, cells were stained with 5 μM BODIPY for 15 min and washed three times with PBS. Cell nuclei were stained with 1 μg/ml DAPI for 15 min, followed by PBS wash for three times. Images were taken with LSCM and the green fluorescence indicated lipid droplets within cells were quantified by ImageJ.

### Stiffness characterization of abdominal subcutaneous at in vivo

The study was conducted in accordance with the Declaration of Helsinki and approved by the Ethics Committee of Tangdu Hospital (No. K202411-19). All subjects provided consent for their data to be used in this study, and the key information about the cohort (*e.g.*, sample size, BMI, age, sex, and metabolic status) was listed in Supplementary Table S1. The stiffness of abdominal subcutaneous AT was characterized by Ultrasound Elastography. This choice is clinically motivated: subcutaneous fat is directly accessible for noninvasive ultrasound measurement without surgical intervention, and abdominal subcutaneous AT stiffness correlates strongly with visceral adiposity accumulation and metabolic dysfunction. With the individuals in supine position and breathing normally, the probe is placed on the right abdomen about 5 cm away from the umbilicus to avoid interference from the liver, spleen, and periumbilical fiber structure. The area with subcutaneous fat layer thickness ≥ 1 cm was selected for measurement, and the average Young’s modulus was automatically calculated once the elastic map was stable. The measurements were repeated three times, and the average value was taken as the final AT stiffness.

### ELISA assay of LOX

The LOX activity of 3T3-L1 cells after cultured on GelMA hydrogels with different stiffness for 3 days was assessed by Mouse LOX ELISA Kit. Briefly, the culture supernatant was collected into aseptic tubes and centrifuged for 20 min at 3000 rpm. Then, serially diluted replicates were incubated with reagent mix, and subsequently absorbance optical density (O.D.) was measured by Microtiter Plate Reader at 450 nm within 15 min. The result was normalized by total protein concentration.

### LOX immunofluorescence staining

Cells were fixed in 4% PFA for 15 min, washed three times in PBS for 5 min each, permeabilized with 0.2% Triton X-100 for 10 min, and washed again three times in PBS for 5 min each. Next, we blocked nonspecific binding with 5% BSA for 60 min. The LOX antibody (Bioworld Technology, BS70865) was diluted 1:200 and incubated at 4°C overnight. The next day, samples were washed with PBS three times for 10 min each to eliminate unbound primary antibodies. Fluorescently labeled secondary antibodies (Bioworld Technology, BS10034) were diluted 1:1000 and incubated in the dark for 1 h. Nuclei were stained with a DAPI solution of 1 μg/ml for 10 min at room temperature. Finally, the samples were washed three times with PBS for 5 min each. Images were taken with LSCM for the observation of LOX expression or localization.

### Quantitative reverse transcription polymerase chain reaction

The primers in the work for qRT-PCR were listed in Supplementary Table S2. The Total RNA was extracted from cultured cells and qRT-PCR assays were performed according to the manufacturer’s protocol. For data analysis, the ΔΔCT method was utilized in Excel.

### Western blotting

3T3-L1 cells were cultured on GelMA hydrogels with different stiffness for 2 days, and then lysed using radioimmunoprecipitation assay buffer with protease inhibitors. The protein concentration of each sample was measured using a BCA assay kit. Equal amounts of total protein (50 μg) from each sample were separated in a 10% SDS-PAGE gel and transferred to a polyvinylidene difluoride membrane at 120 V for 2 h at room temperature. The blot was blocked with 3% nonfat dry milk suspended in TBS-T for 1 h at room temperature. Membranes were incubated with primary antibodies overnight at 4°C. The resulting blots were incubated with secondary antibodies (anti-rabbit IgG HRP-linked antibody) at 1:3000 dilution. Bands were scanned using a densitometer (Bio-Rad) and quantified using the Quantity One 4.6.3 software (Bio-Rad).

### Animal model construction and treatment

The experiments were approved by the Xi’an Jiaotong University Biomedical Ethics Committee (No. 2025-124). 8-weeks male C57BL/6J mice were used to establish Control, Weight gain, Obesity and Weight rebound model through dietary adjustments. The Control group was consistently fed normal diet (ND). The Weight gain group was fed ND in the early stage and fed high-fat diet (HFD) in the later stage. The Obesity group was continuously fed HFD. The Weight rebound group was fed HFD until the Weight gain exceeded 20% of the initial weight, and then switched to ND until the weight was similar to the Control group, and finally switched to HFD. The body weights were regularly recorded during the feeding process. *In vivo* treatment: BAPN (100 mg/kg/day) was dissolved in drinking water and administered to mice at the beginning of the weight-rebound protocol and continued throughout the obesity, weight-loss, and rebound stages, the Control group received the equivalent volume of plain drinking water. and the volume of water consumed was monitored daily to confirm consistent dosing.

### Body fat analysis

After feeding, the fat content of the mice was tested using a body composition analyzer (Bruker, LF90, Germany), and the body fat percentage was calculated. Body fat percentage (%) = fat weight/body weight × 100%.

### Glucose metabolism

After fasting overnight, the glucose metabolism function was evaluated by intraperitoneal glucose tolerance test. Inject 2 g/kg glucose solution intraperitoneally, and collect blood samples by cutting the tail after alcohol disinfection before glucose injection (0 min), 15, 30, 60, 120, and 180 min after injection. Use a blood glucose meter to detect blood glucose and record the values.

### Lipid metabolism

After fasting for 12 h, blood was collected through the retroorbital vein. Centrifuge the blood sample at 4°C for 10 min (3000 rpm/min), and take the upper serum for ELISA to determine total cholesterol (TC), triglycerides (TG), high-density lipoprotein (HDL), and low-density lipoprotein (LDL).

### Stiffness measurement of *ex-vivo* eWAT

All animal experiments use epididymal adipose tissue (eWAT)—a visceral adipose depot. We chose eWAT because it is the most extensively characterized visceral fat depot in male C57BL/6J mice, shows the most pronounced fibrotic and inflammatory changes under HFD feeding, and is the most metabolically active fat depot with the greatest impact on systemic insulin resistance. *Ex-vivo* eWAT of mice were sliced into frozen sections with a thickness of 100 μm, and the stiffness was measured by atomic force microscopy (AFM). Find the field of view of AT under the microscope, run AFM operation software, and use a spherical probe to measure through contact mode. The probe scanning range is 125 μm × 125 μm, using a 200 μm microcantilever with an elastic constant velocity of 0.09 N/m. The image is obtained in constant force mode. All images are automatically smoothed to eliminate noise, and the stiffness values of AT are obtained through calculation and analysis of the measured data.

### Masson staining for collagen quantification

After dewaxing with xylene and ethanol, the paraffin sections of AT were stained with hematoxylin and masson in sequence, dehydrated, and finally sealed with neutral gum. Observe and photograph the staining results under a microscope, and quantify the collagen content using ImageJ software.

### Immunohistochemical (IHC) staining

Slides were first hydrated, antigen retrieval, incubated with primary antibody (LOX, Bioworld Technology, BS70865, USA) at 4°C overnight. And then incubated with secondary antibody at 37°C for 20 min. After washing, slides were developed with DAB-Chromogen for 8 min followed by hematoxylin/eosin (H&E) counterstaining.

### Statistical analysis

Statistical analysis was conducted using Origin Pro 2016 (USA). Data are presented as mean ± standard deviation (SD). For two-group comparisons, unpaired two-tailed Student’s *t* test was used; and for multigroup independent comparisons, one-way ANOVA with Tukey’s post hoc test was applied. For the analysis of longitudinal repeated measurements with two factors (group × time), we applied a linear mixed-effects model with group, time, and their interaction as fixed effects and subject-specific random intercepts. This approach was chosen for its robustness against missing data and its ability to model appropriate covariance structures without the sphericity assumption required by traditional repeated-measures ANOVA. Post-hoc pairwise comparisons were adjusted using Tukey’s HSD test. Correlation analysis was performed using Pearson’s correlation. (n.s., no significance; ∗*P* < 0.05; ∗∗*P* < 0.01; ∗∗∗*P* < 0.001).

## Results

### AT stiffening emerges in obesity and correlates with high expression of LOX in clinical samples

**Fig. 1:**
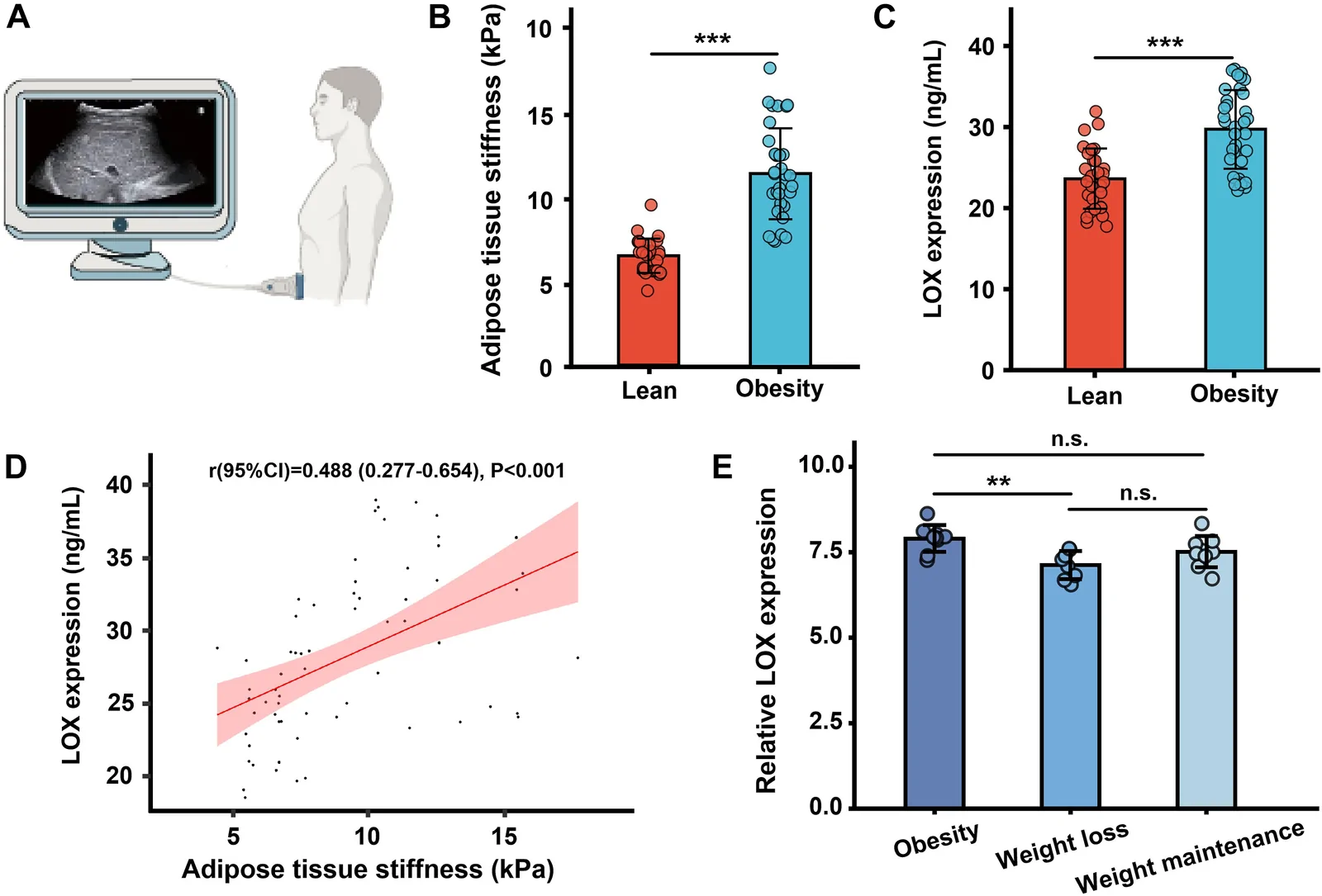
AT stiffening emerges in obesity and correlates with high expression of LOX in clinical samples. A: Schematic diagram of abdominal subcutaneous AT stiffness measurement. B: AT stiffness of lean and obese patients measured by ultrasonic elastography. C: LOX expression in blood serum of lean and obese patients. D: Correlation analysis between abdominal subcutaneous AT stiffness and LOX expression. E:) LOX expression in subcutaneous AT of obese patients during obesity, weight loss, and weight maintenance (data source: http://www.ncbi.nlm.nih.gov/geo/GSE35411). (n.s., no significance; ∗*P* < 0.05; ∗∗*P* < 0.01; ∗∗∗*P* < 0.001). AT, Achilles tendon; LOX, lysyl oxidase.

To determine whether AT mechanical properties are altered in lean and obese individuals, we characterized the stiffness of subcutaneous AT in the abdomen of these two populations by ultrasound elastography technology (Fig. 1A). We observed that AT stiffness in obese population was significantly higher than that in lean controls (Fig. 1B). Consistent with the mechanical alterations, serum LOX levels were markedly upregulated in the obese cohort (Fig. 1C). We further performed Pearson’s correlation analysis between stiffness and LOX expression, and found a significant positive correlation between these two variables (Pearson r = 0.488, 95% CI: 0.277–0.654, *P* < 0.001) (Fig. 1D), confirming that higher AT stiffness is directly associated with elevated serum LOX levels at the individual subject level. Furthermore, we analyzed LOX expression in subcutaneous abdominal AT from obese individuals in database, and observed significantly decreased LOX expression after weight loss but rebounded again during the weight maintenance stage, with no significant difference from the initial obesity state (Fig. 1E). Collectively, these clinical findings establish a robust triad linking obesity, tissue stiffness, and LOX upregulation. Crucially, the recurrence of high LOX levels during weight maintenance points to a persistent fibrotic signature, suggesting that the LOX-stiffness axis may serve as a latent driver of weight rebound.

### LOX-mediated at stiffness change promotes weight rebound

**Fig. 2:**
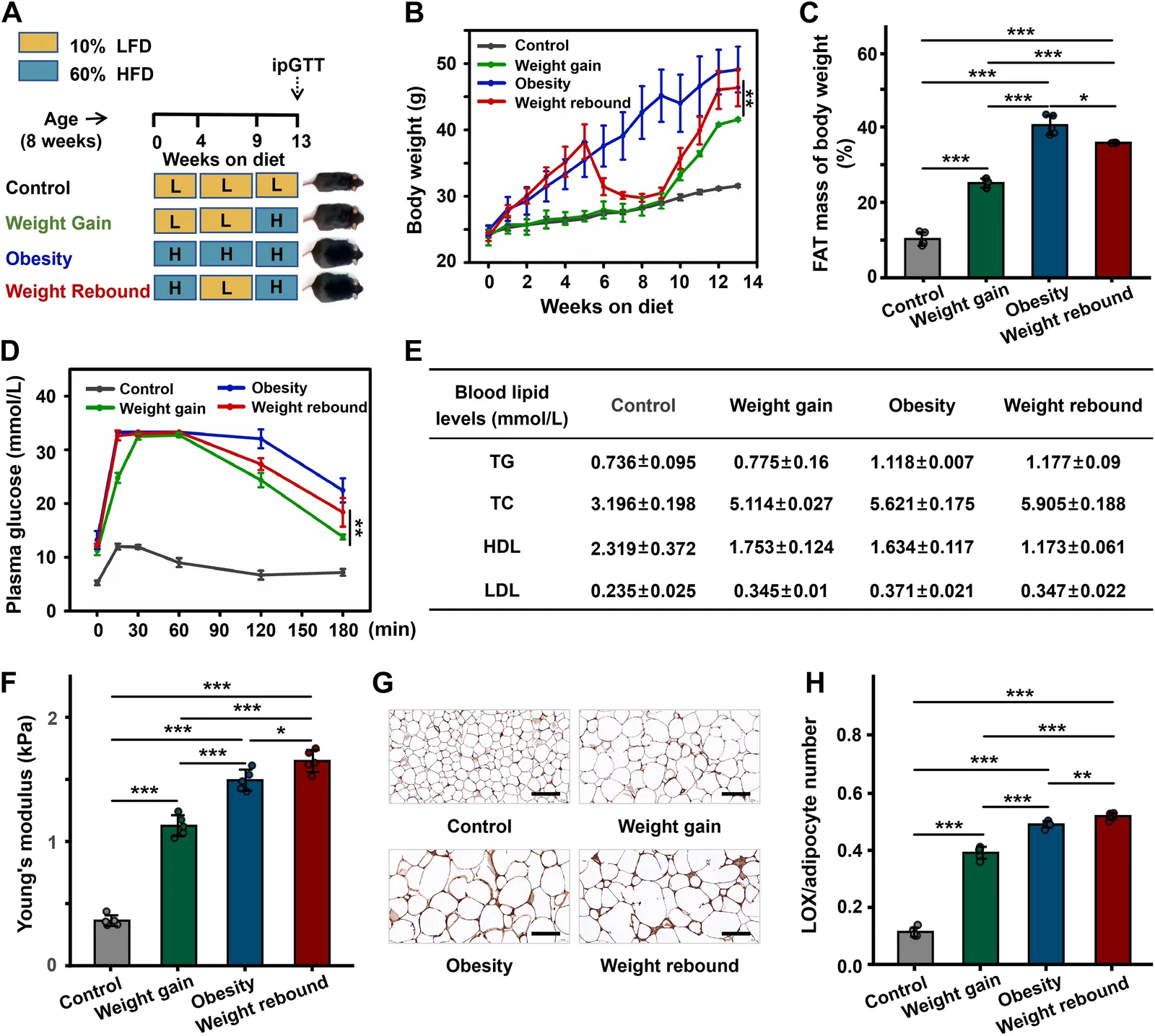
Construction of animal models (Control, Weight gain, Obesity, and Weight rebound group) and metabolic analysis. A: Animal models feeding plan. B: Body weight change curve of different groups. Data are presented as mean ± SD (n = 5). C: Quantitative analysis of body fat percentage. Data are presented as mean ± SD (n = 5). D: Mouse peritoneal glucose tolerance test (upper limit of blood glucose detected by blood glucose meter is 33.3 mmol/L) Data are presented as mean ± SD (n = 5). E: Results of blood lipid metabolism. Data are presented as mean ± s.d. (n = 5). F: Quantification of AT stiffness measured by AFM. Data are presented as mean ± s.d. (n = 5). G: Immunohistochemical staining. H: Quantitative analysis of LOX in epididymal AT across all groups (the scale bar represents 150 μm). Data are presented as mean ± SD (n = 5). (n.s., no significance; ∗*P* < 0.05; ∗∗*P* < 0.01; ∗∗∗*P* < 0.001). AFM, atomic force microscopy; HDL: High density lipoprotein cholesterol; LDL: Low density lipoprotein cholesterol; LOX, lysyl oxidase; TC: Total cholesterol; TG: Triglycerides.

To validate the clinical observations, we established a diet-induced mouse model recapitulating obesity and different stages of weight rebound (Fig. 2A). After weight loss, mice showed significantly faster weight rebound and higher body fat percentage when re-fed with HFD compared to the Control group (Fig. 2B, C). This indicates that adipocytes might be “pre-programmed” after experiencing obesity, and are more sensitive to signals of over-nutrition, leading to faster weight rebound. Further intraperitoneal glucose tolerance test and lipid analysis showed that metabolic function of weight rebound mice was significantly worse than that of the Control and Weight gain group, and closer to the long-term Obesity group (Fig. 2D, E and Supplementary Fig. S1). Besides, in order to examine whether AT stiffness and LOX expression differ among the four experimental groups, we further measured AT stiffness using AFM and analyzed LOX expression in epididymal AT. It was observed that AT stiffness progressively increased from the Control group to the Weight gain, Obesity, and Weight rebound groups (Fig. 2F), the immunohistochemical staining and subsequent quantitative analysis revealed a similar increasing trend for LOX expression, with the highest levels observed in the Weight rebound group (Fig. 2G, H). These results preliminarily indicated that weight rebound may be related to high AT stiffness and high LOX expression.

**Fig. 3:**
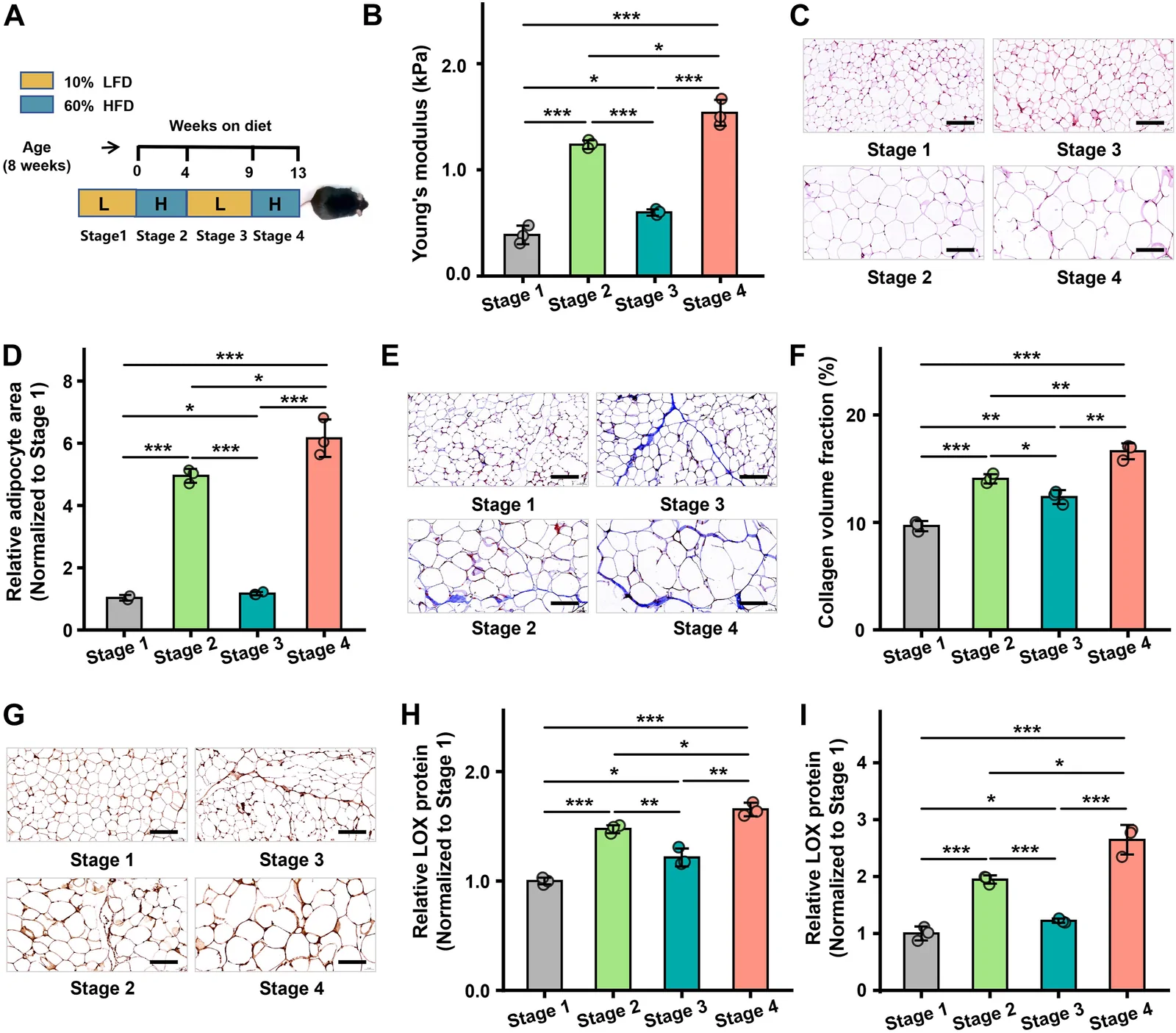
AT stiffness dynamically changes in different stages in Weight rebound model and is consistent with the trend of LOX changes. A: The construction of Weight rebound model and the explanation of different stages (Stage 1: Initial stage; Stage 2: Obesity stage; Stage 3: Weight loss stage; Stage 4: Rebound stage). B: Quantification of AT stiffness measured by AFM. Data are presented as mean ± SD (n = 3). C: HE staining. D: Quantification of adipocyte size (the scale bar represents 150 μm). Data are presented as mean ± SD (n = 3). E: Masson staining. F: Quantification of collagen content results (the scale bar represents 150 μm). Data are presented as mean ± SD (n = 3). G: Immunohistochemical staining. H: Quantitative analysis of LOX in epididymal AT of mice (the scale bar represents 150 μm). Data are presented as mean ± SD (n = 3). I: Western blotting analysis of LOX proteins in epididymal AT of mice at different stages. Data are presented as mean ± SD (n = 3). (n.s., no significance; ∗*P* < 0.05; ∗∗*P* < 0.01; ∗∗∗*P* < 0.001). AFM, atomic force microscopy; AT, Achilles tendon; LOX, lysyl oxidase.

Next, we plotted the overall mechanical changes in adipose tissue behind weight rebound. The AFM result showed that AT stiffness dynamically changes in different stages of weight rebound, HE and Masson staining were performed to explain the phenomenon (Fig. 3A–F). Specifically, AT stiffness significantly increases during the obesity period, which mainly due to adipocyte hypertrophy and excessive collagen deposition. During the weight loss period, AT stiffness showed a decreasing trend but did not fully normalize, as some collagen cross-linking and fibrosis were irreversible, although the difference from the initial stage did not reach statistical significance. It is noteworthy that in the rebound stage (Stage 4), AT stiffness not only increases with weight rebound but also tends to exceed the levels observed in the obesity stage (Stage 2), although the difference between Stage 2 and Stage 4 did not reach statistical significance with the current sample size (Fig. 3B).

We further analyzed the dynamic changes of LOX expression in the AT of mice. Consistent with mechanical data, LOX expression exhibits persistent elevation (Fig. 3G–I and Supplementary Fig. S2). Specifically, quantitative analysis showed that the expression of LOX remained elevated after weight loss compared to the initial stage (Fig. 3I), indicating the presence of residual pro-fibrotic signals in AT. During the rebound stage, LOX expression showed an increasing trend, consistent with the trend in AT stiffness. Overall, these results suggest that LOX persistence may form the basis for the observed mechanical hysteresis, which may maintain a mechanical microenvironment favorable for rapid weight rebound.

### LOX links matrix stiffness to lipid accumulation

**Fig. 4:**
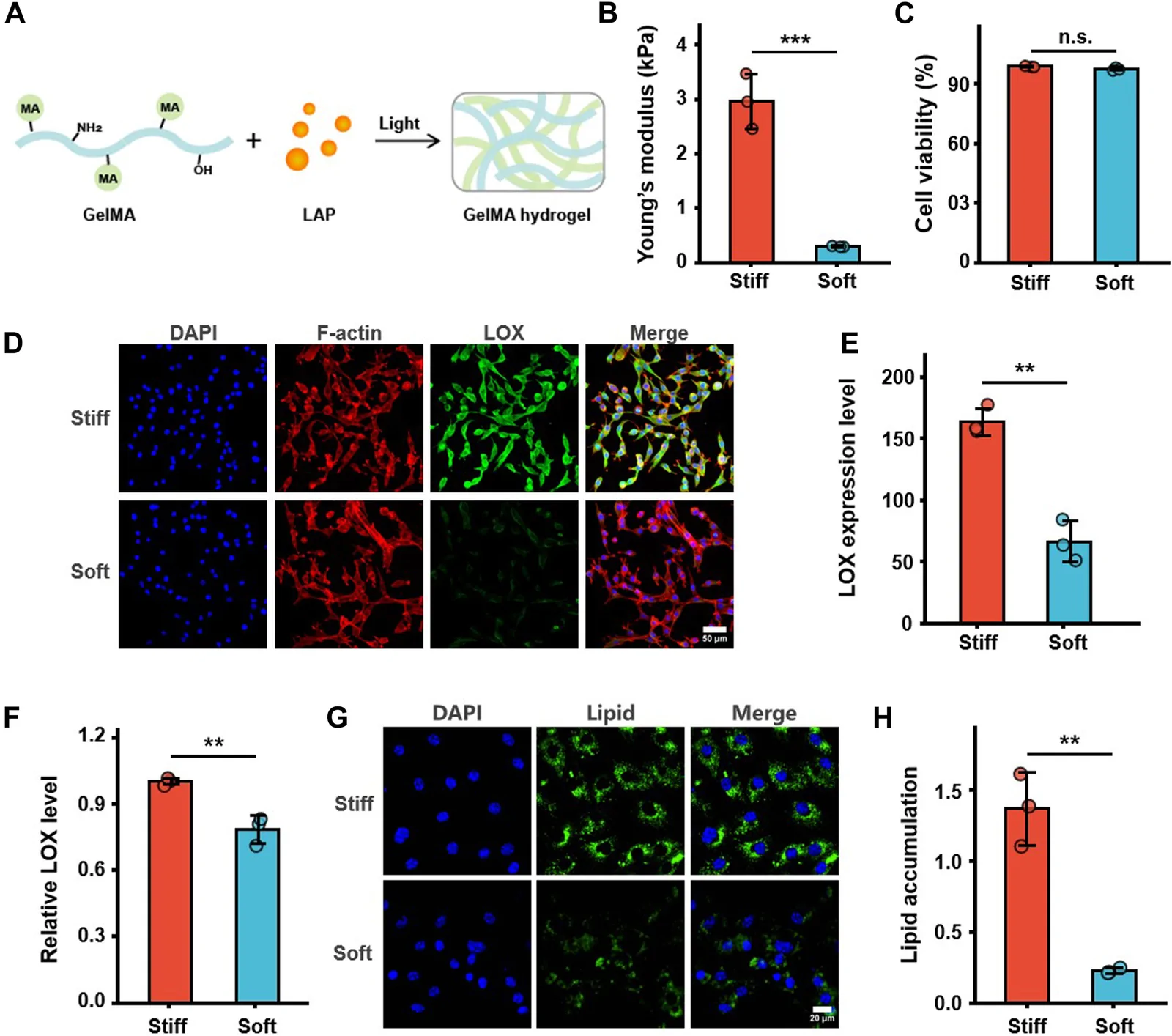
Matrix stiffness induces high LOX expression and increased lipid accumulation. A: Schematic diagram of GelMA hydrogel preparation. B: Hydrogel stiffness tested by rheometer. Data are presented as mean ± SD (n = 3). C: Cell viability of 3T3-L1 cells after 24 h culturing on the hydrogels. Data are presented as mean ± SD (n = 3). D: Immunofluorescence staining of LOX in 3T3-L1 cells after culturing on the hydrogels with different stiffness (the scale bar represents 50 μm). E: Quantification of LOX level from D. Data are presented as mean ± SD (n = 3). F: Secreted LOX level measured by ELISA. Data are presented as mean ± SD (n = 3). G: BODIPY staining for differentiation of 3T3-L1 cells after culturing on the hydrogels with different stiffness (the scale bar represents 20 μm). H: Quantification of lipid accumulation from G. Data are presented as mean ± SD (n = 3). (n.s., no significance; ∗*P* < 0.05; ∗∗*P* < 0.01; ∗∗∗*P* < 0.001). GelMA, methacryloyl gelatin; LOX, lysyl oxidase.

To explore the role of matrix stiffness in a controlled in vitro system, we prepared GelMA hydrogel with different stiffness to simulate the mechanical microenvironment of adipocytes during weight rebound (Fig. 4A). While fibroblasts and other stromal cells are the predominant cellular sources of LOX in vivo, our 3T3-L1-based in vitro system was designed to investigate whether adipocytes themselves exhibit mechanosensitive LOX upregulation that could amplify the pro-adipogenic response to matrix stiffening. As the stiffness of subcutaneous AT in the clinical cohort is affected by many factors (*e.g.*, age, sex, metabolic status, etc.), we selected the AT stiffness changes in different stages of weight rebound mice as the stiffness reference in vivo and prepared hydrogels with stiffness ranging from ∼0.3 kPa to ∼3 kPa, with good biocompatibility (Fig. 4A, B and Supplementary Fig. S3). The results of immunofluorescence (IF) staining and ELISA test both showed that LOX was higher expressed in 3T3-L1 cells under high stiffness (Fig. 4D–F and Supplementary Fig. S4). Meanwhile, it was observed higher lipid accumulation in 3T3-L1 cells after culture on high matrix stiffness. These results indicate that high matrix stiffness can not only promote lipid accumulation, but also significantly upregulate endogenous LOX expression, which may further strengthen the fibrotic microenvironment.

**Fig. 5:**
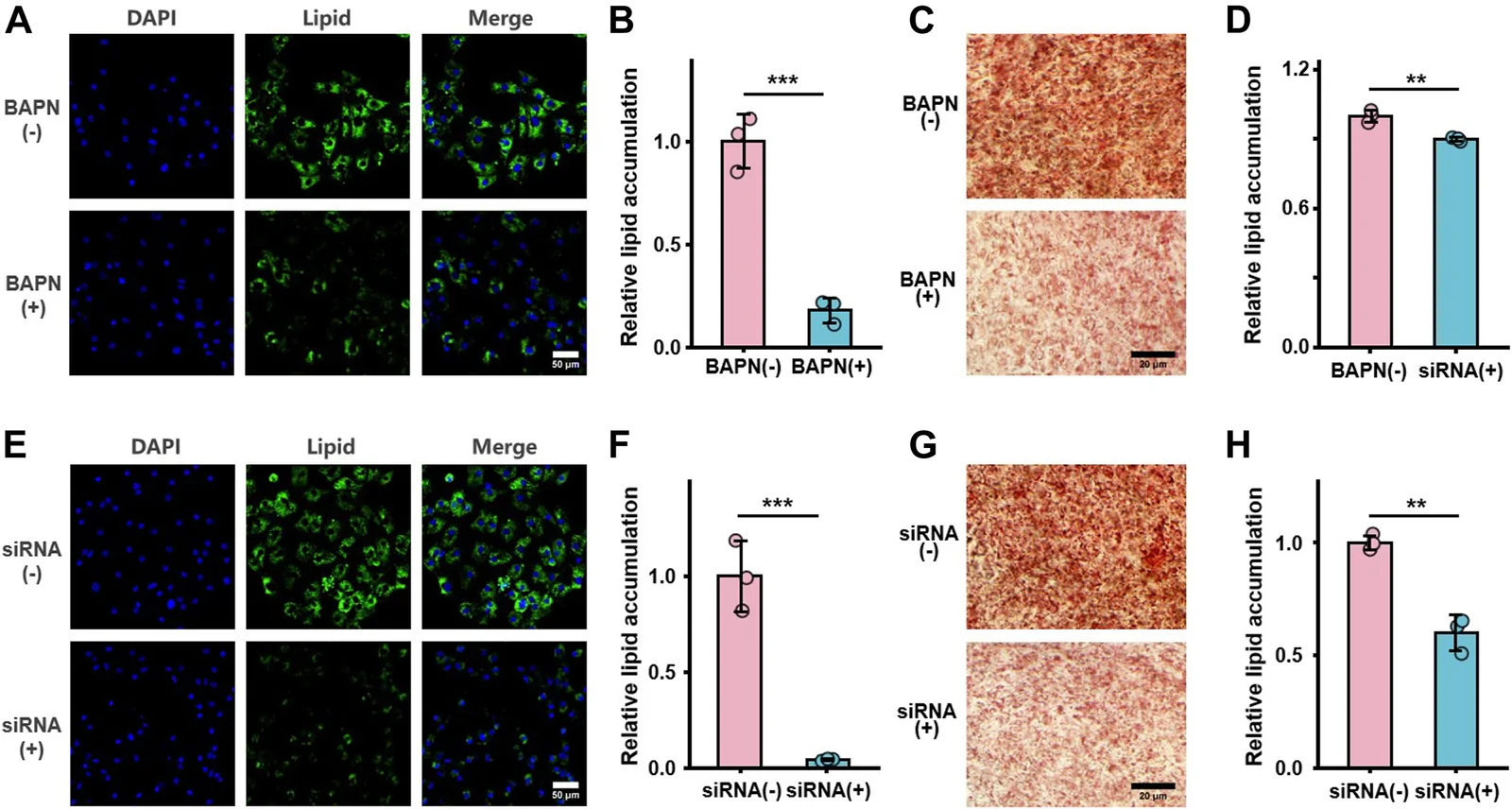
LOX inhibition/knockdown impairs lipid accumulation ability of 3T3-L1 cells on stiff matrix (∼3 kPa). A: BODIPY staining for differentiation of 3T3-L1 cells with/without BAPN inhibition on stiff matrix (the scale bar represents 50 μm). B: Quantification of lipid accumulation from A. Data are presented as mean ± SD (n = 3). C: Oil-red staining for differentiation of 3T3-L1 cells with/without BAPN inhibition on stiff matrix (the scale bar represents 20 μm). D: Quantification of oil-red staining for differentiation of 3T3-L1 cells with/without BAPN inhibition on stiff matrix. Data are presented as mean ± SD (n = 3). E: BODIPY staining for differentiation of 3T3-L1 cells with/without siRNA knockdown on stiff matrix (the scale bar represents 50 μm). F: Quantification of lipid accumulation from E. Data are presented as mean ± SD (n = 3). G: Oil-red staining for differentiation of 3T3-L1 cells with/without siRNA knockdown on stiff matrix (the scale bar represents 20 μm). H: Quantification of oil-red staining for differentiation of 3T3-L1 cells with/without siRNA knockdown on stiff matrix. Data are presented as mean ± SD (n = 3). (n.s., no significance; ∗*P* < 0.05; ∗∗*P* < 0.01; ∗∗∗*P* < 0.001). BAPN, β-aminopropionitrile; LOX, lysyl oxidase.

To further clarify the relationship between LOX expression and lipid accumulation, we studied the lipid accumulation level of 3T3-L1 cells after LOX inhibition with BAPN and LOX knockdown with siRNA on different matrix stiffness. We firstly examined the cytotoxicity in 3T3-L1 cells in vitro using methyl thiazolyl tetrazolium assay to determine the optimal concentration of BAPN for inhibiting LOX activity, and observed that cell viability was not affected by 1 mM BAPN (Supplementary Fig. S5). And then, 1 mM was selected as the optimal concentration of BAPN for LOX inhibition, and BODIPY and oil-red staining were performed to evaluate the lipid accumulation ability of 3T3-L1 cells. It was observed that lipid accumulation in 3T3-L1 cells significantly decreased when the LOX was inhibited with BAPN on stiff matrix (∼3 kPa) (Fig. 5A–D). Furthermore, BODIPY and oil-red staining were also performed in 3T3-L1 cells after treatment with/without siRNA listed in Supplementary Table S3, and lower lipid accumulation was observed after LOX knockdown (Fig. 5E–H and Supplementary Fig. S6). Finally, the same LOX intervention experiments (including LOX inhibition and knockdown) and staining experiments (including BODIPY and oil-red staining) were performed in 3T3-L1 cells on soft matrix (∼0.3 kPa), but observed no significant difference (Supplementary Fig. S7). These results indicate that the pro-adipogenic role of LOX appeared more evident on the stiff matrix, both inhibiting LOX activity and reducing its expression on stiff matrix can effectively suppress adipogenesis. Therefore, LOX may be a potential target in the management of obesity or weight rebound.

### LOX inhibition slows down the weight rebound process in vivo

**Fig. 6:**
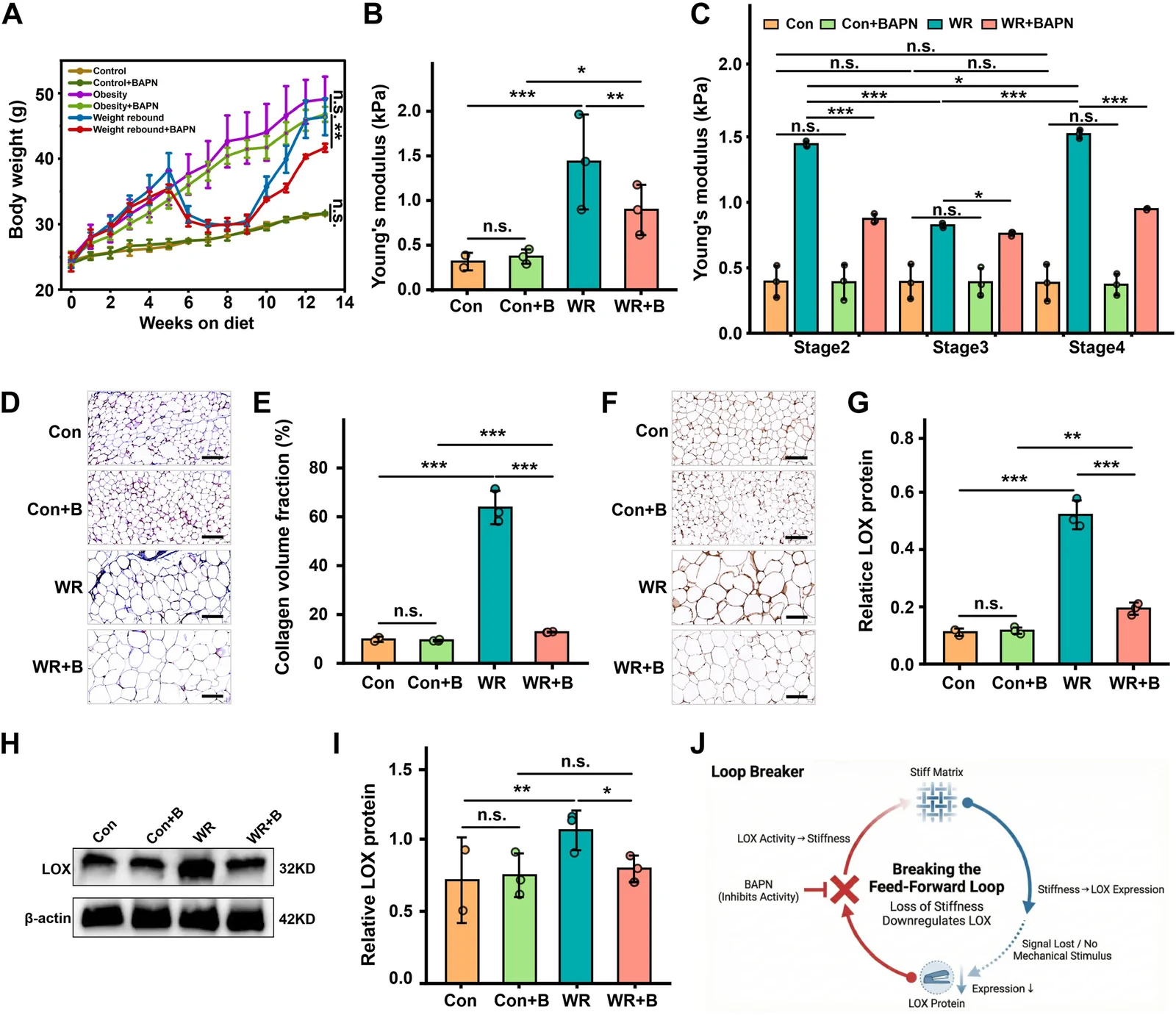
LOX inhibition slows down the weight rebound process in vivo. A: Mouse weight change curve. Data are presented as mean ± SD (n = 3). B: AT stiffness (Con: Control; WR: Weight Rebound; and +B: +BAPN). Data are presented as mean ± SD (n = 3). C: AT stiffness at different stages (Stage 2: Obesity stage; Stage 3: Weight loss stage; and Stage 4: Rebound stage) in the Control and Weight rebound group (Con: Control; WR: Weight Rebound; and +B: +BAPN). Data are presented as mean ± SD (n = 3). D: Masson staining of AT slices (the scale bar represents 150 μm). E: Quantitative results of collagen content. Data are presented as mean ± SD (n = 3). F: LOX immunohistochemical staining of AT slices (the scale bar represents 150 μm). G: LOX quantification results. Data are presented as mean ± SD (n = 3). H: Western blotting analysis. I: quantification of LOX proteins in epididymal adipose tissue of mice after treatment with/without BAPN. Data are presented as mean ± SD (n = 3). (n.s., no significance; ∗*P* < 0.05; ∗∗*P* < 0.01; ∗∗∗*P* < 0.001) J: Schematic of the “Loop Breaker” mechanism. The diagram illustrates that pharmacological inhibition of LOX activity (red cross) leads to matrix softening. This loss of stiffness removes the mechanical stimulus required to maintain high LOX expression (“Signal Lost”), resulting in a secondary downregulation of LOX protein. AT, Achilles tendon; LOX, lysyl oxidase.

To investigate the effect of LOX on weight rebound in vivo, BAPN (100 mg/kg/day) was added into the drinking water of mice to inhibit LOX activity. And the weight change curve showed that BAPN could slow down the process of weight rebound, but without an obvious effect on body weight in the Control and Obesity group under the present experimental conditions (Fig. 6A). To further demonstrate the mechanism of LOX inhibition in slowing down weight rebound, we firstly characterized AT stiffness at different stages by using AFM. The data revealed that BAPN treatment begins to reduce AT stiffness as early as the obesity stage (Stage 2), and the divergence between treated and Control groups becomes more pronounced during the rebound stage (Stage 4), confirming that continuous BAPN treatment progressively disrupts the fibrotic cycle (Fig. 6B, C). Meanwhile, this restoration of mechanical compliance is accompanied by the normalization of collagen deposition (Fig. 6D, E). Notably, we also observed a significant downregulation of LOX protein levels (Fig. 6F–I). This finding suggests that the decrease of AT stiffness successfully disrupts the cycle of “stiffness induced LOX expression” (Fig. 6J), leading to the interruption of LOX induced fibrosis signals and ultimately slowing down weight rebound.

## Discussion

At present, the understanding on weight rebound mainly focus on physiological driving factors, including AT inflammation, reduced energy expenditure, reduced oxidative decomposition and changes of appetite related hormones (11). Our study introduces a biomechanical perspective that complements traditional views of weight rebound. We propose and provide evidence for a LOX-stiffness axis as a central regulator. This axis exhibits a distinct trajectory characterized by accumulating clinical evidence: AT stiffness rises sharply during obesity, incompletely resolves post-weight loss, and escalates rapidly upon regain. This persistent elevation of stiffness may represent a persistent biomechanical alteration that creates a pre-programmed mechanical microenvironment, making adipocytes highly sensitive to nutritional signals and prone to weight rebound.

An important consideration in interpreting our findings is the distinction between the adipose depots studied. It is well established that subcutaneous and visceral adipose depots differ profoundly in their cellular composition, inflammatory milieu, ECM architecture, and remodeling capacity. Visceral fat, particularly eWAT, harbors a higher density of macrophages and pro-inflammatory crown-like structures, exhibits greater collagen cross-linking and TGF-β-driven fibrotic signaling, and is more directly linked to systemic insulin resistance and metabolic disease compared to SAT (27, 28). Accordingly, visceral fat is considered the "metabolically active" depot, while subcutaneous AT exerts a more protective buffering role under conditions of caloric surplus (29). Despite these structural differences, our cross-depot data reveal a consistent pattern: both subcutaneous AT (clinical) and eWAT (animal) show LOX upregulation and elevated stiffness in obesity. This concordance points to a depot-independent mechanism in which LOX-mediated irreversible collagen cross-linking creates a persistent fibrotic scaffold that resists normalization after weight reduction. We therefore propose that the LOX-stiffness axis represents a broadly operative pro-adipogenic mechanism, albeit one that may be most pronounced in metabolically active visceral depots. Future studies directly comparing paired subcutaneous AT and visceral AT biopsies from patients undergoing structured weight loss and regain programs, will be important next steps to fully delineate the depot-specific contribution to weight rebound.

In fact, the mechanical properties of the AT microenvironment may not act in isolation to induce weight rebound, but in synergy with the metabolic factors. We confirm that pathological AT stiffening is not only a consequence of obesity, but also a key codriver of physiological dysfunction. For instance, high ECM stiffness can activate pro-inflammatory signals (*e.g.*, via NF-κB and mechanosensitive YAP/TAZ) in adipocytes (30, 31), while inflammatory cytokines such as TGF-β in turn stimulate LOX mediated cross-linking, exacerbating tissue fibrosis and stiffness (32, 33). This creates a self-reinforcing “mechano-inflammatory” cycle, linking mechanical cues to metabolic inefficiency (34, 35), explaining why addressing inflammation alone often cannot prevent weight rebound.

Mechanistically, we propose and provide evidence for a LOX-stiffness axis as a central regulator. This mechanism is rooted in the LOX-catalyzed covalent cross-linking of collagen within the ECM, a biochemical process directly engineering tissue stiffening (36, 37). Our clinical evidence solidifies this link: obesity is associated with elevated AT stiffness and heightened serum LOX expression in human subjects (Fig. 1). Crucially, in animal models, LOX expression mirrors the dynamic stiffness trajectory, peaking during weight rebound, and pharmacological LOX inhibition with BAPN markedly attenuates stiffness and slows rebound, supporting a functional contribution of the LOX-stiffness axis. Therefore, mechanotherapy targeting the mechanical properties of AT represents a unique and necessary therapeutic dimension beyond traditional metabolic interventions. In this study, the in vitro experiments were performed using 3T3-L1 preadipocytes, which do not represent the full complexity of the AT microenvironment in vivo. In mature adipose tissue, LOX is predominantly expressed by fibroblasts and stromal cells, rather than by differentiated adipocytes. Our adipocyte-focused approach was designed to isolate the mechanical response of adipocytes to matrix stiffness, but does not capture the full ecosystem of LOX-producing cells in vivo. In the future, it will be necessary to study the contributions of LOX to weight rebound through the co-culture experiment of fibroblasts and adipocytes, which simulates physiological environments more realistically.

As a key enzyme involved in ECM remodeling and AT fibrosis, LOX is intrinsically linked to chronic inflammation (14), and the two may synergize to promote weight rebound. We found that LOX is a promising therapeutic target for long-term weight management, but the translational potential of systemic LOX inhibition is currently limited. On the one hand, it is difficult to determine the critical window of LOX inhibition during weight rebound process (including obesity stage, weight loss stage and rebound stage). On the other hand, although LOX inhibition with BAPN effectively slowed down the speed of weight rebound in vivo, BAPN is a nonspecific small molecule with significant systemic toxicity and off-target effects (38, 39, 40). This will lead to the dilemma of treatment facing the possibility of overlapping effective doses with toxic doses, thus more precise methods must be adopted. The latest advances in nanotechnology provide a convincing solution. The engineered nanocarriers can specifically deliver therapeutic agents to adipocytes to improve therapeutic efficacy (41). Looking forward, we have envisioned the development of mechano-nanomedicine targeting the disruption of the “pre-programmed” mechanical microenvironment of adipocytes. By combining biomechanical targets such as LOX with targeted delivery systems, it is expected to transform weight management from a passive struggle against rebound to an active targeted intervention.

Our study suggests AT stiffness as a potential noninvasive biomarker for rebound risk. Although our clinical data provide initial impetus for this hypothesis, we acknowledge the limitations of the current sample size. In the following research, we will expand the cohort and establish a comprehensive “AT stiffness database” to analyze the mechanical characteristics of individuals at their different stages of weight rebound. This lays the foundation for integrating artificial intelligence (AI) into precise obesity management. Just as current AI models can be used to predict weight trajectories based on clinical metadata (42, 43, 44, 45), future algorithms can incorporate “biomechanical biomarkers” (such as AT stiffness scores, LOX levels) to identify high-risk individuals with rapid rebound. This data-driven approach will allow clinicians to stratify and screen patients who may benefit specifically from the antifibrotic mechanical therapy proposed below.

## Conclusion

In summary, our study identifies mechanical “pre-programmed” of AT as a previously overlooked driver in weight rebound and proposes a mechanobiological axis linking AT stiffness, LOX and lipid accumulation. We demonstrated that obesity induced LOX overexpression creates a persistent rigid scaffold, forming a “pre-programmed” mechanical microenvironment that stimulates adipocytes for rapid lipid re-accumulation and further weight rebound. Importantly, we provided in vivo validation that LOX intervention can effectively attenuate AT stiffening, disrupt the pro-fibrotic feedforward loop, and slow down weight rebound. Overall, these findings highlight matrix stiffness as a key therapeutic target and propose anti-fibrotic mechanotherapy as a potential strategy to break the cycle of obesity recurrence.

## Ethics approval and consent to participate

The animal experiments were approved by the Xi’an Jiaotong University Biomedical Ethics Committee (No. 2025-124). The stiffness characterization of abdominal subcutaneous AT in vivo was conducted in accordance with the Declaration of Helsinki and approved by the Ethics Committee of Tangdu Hospital (No. K202411-19).

## Data availability

The datasets used and/or analyzed during the current study are available from the corresponding author on reasonable request.

## Supplemental data

This article contains supplemental data.

## Conflicts of interest

The authors declare that they have no conflicts of interest with the contents of this article.
