# Supplementary material for: LOX-mediated adipose tissue stiffening promotes weight rebound
Source: J Lipid Res. 2026 Aug 11;67(9):101121. doi: 10.1016/j.jlr.2026.101121 (PMC13571246; doi:10.1016/j.jlr.2026.101121)
Supplement: Supplementary file 1 [file mmc1.docx]

**SUPPLEMENTARY INFORMATION**

**LOX-mediated Adipose Tissue Stiffening Promotes Weight Rebound**

**Supplementary Figure S1. Blood lipid metabolism analysis.** Blood lipid analysis of **(a)** Triglycerides (TG), **(b)** Total cholesterol (TC), **(c)** Low density lipoprotein cholesterol (LDL) and **(d)** High density lipoprotein cholesterol (HDL). Data are presented as mean±s.d. (n=5). (n.s., no significance; **P* < 0.05; ***P* < 0.01; ****P* < 0.001).

**Supplementary Figure S2.** Western blotting analysis of LOX proteins in epididymal AT of mice at different stages.

**Supplementary Figure S3.** Live-dead cell staining of 3T3-L1 cells after culture on hydrogel for 24 hours (scale bar: 200 μm). (Green, live cells; red, dead cells).

**Supplementary Figure S4.** Standard curve of LOX assessed by Mouse Lysyl Oxidase (LOX) ELISA Kit.

**Supplementary Figure S5.** Cell viability of 3T3-L1 cells after 48 h incubation with different concentrations of BAPN. Data are presented as mean±s.d. (n=3).

**Supplementary Figure S6.** **(a)** Relative expression of LOX in 3T3-L1 cells after siRNA knockdown. Data are presented as mean±s.d. (n=3). **(b)** Western blotting analysis of LOX proteins with anti-LOX antibody from intact transfected 3T3-L1 cells analyzed by blotting. **(c)** Quantification of LOX proteins from b. Data are presented as mean±s.d. (n=3). (n.s., no significance; **P* < 0.05; ***P* < 0.01; ****P* < 0.001).

**Supplementary Figure S7.** **LOX inhibition/knockdown hardly affects lipid accumulation ability of 3T3-L1 cells on soft matrix (~0.3 kPa).** **(a)** Oil-red staining for differentiation of 3T3-L1 cells with/without BAPN inhibition on soft matrix (scale bar: 20 μm). **(b)** Quantification of oil-red staining for differentiation of 3T3-L1 cells with/without BAPN inhibition on soft matrix. Data are presented as mean±s.d. (n=3). **(c)** Oil-red staining for differentiation of 3T3-L1 cells with/without si-RNA knockdown on soft matrix (scale bar: 20 μm). **(d)** Quantification of oil-red staining for differentiation of 3T3-L1 cells with/without si-RNA knockdown on soft matrix. Data are presented as mean±s.d. (n=3). (n.s., no significance; **P* < 0.05; ***P* < 0.01; ****P* < 0.001)

**Supplementary Table S1.** Clinical characteristics of lean and obese participants.

| **Variable** | **Lean（BMI < 28）** | **Obesity（BMI ≥ 28）** | ***P* Value** |
| --- | --- | --- | --- |
| **n** | 30 | 35 |  |
| **Sex (male/female)** | 9/21 | 14/21 |  |
| **BMI** | 23.49 ± 2.58 | 32.31 ± 3.06 | <0.001 |
| **Age** | 49 ± 10.26 | 43.29 ± 10.53 | 0.03 |
| **Glycosylated hemoglobin (%)** | 5.08 ± 0.45 | 5.19 ± 0.41 | 0.31 |
| **Fasting blood glucose (mmol/L)** | 5.04 ± 0.38 | 5.52 ± 0.66 | <0.001 |

**Supplementary Table S2.** Sequence data of Primers for qRT-PCR.

| **Primer** | **Primer sequence (5'--3'）** |
| --- | --- |
| LOX-F | CACTGCACACACACAGGGAT |
| LOX-R | ATTGTGCAGCCTGAGGCATA |
| B2M-F | TTCTGGTGCTTGTCTCACTGA |
| B2M-R | CAGTATGTTCGGCTTCCCATTC |

**Supplementary Table S3.** Sequence data of primers and LOX siRNA duplexes corresponding to the sequences.

|  | **Sequence (5'--3'）** |
| --- | --- |
| si-LOX1 | ACAAGUACUCCGACGACAA |
| si-LOX2 | GGGCGGAUGUCAGAGACUA |
| si-LOX3 | GAUUGAUAUUACAGAUGUA |
